# NED-oriented oncology in metastatic breast cancer: a hypothesis-driven framework integrating the tumor immune microenvironment, antibody-drug conjugates, and metastasis-directed therapy

**DOI:** 10.3389/fonc.2026.1895475

**Published:** 2026-08-18

**Authors:** Shinichiro Kashiwagi

**Affiliations:** Department of Breast Surgical Oncology, Osaka Metropolitan University Graduate School of Medicine, Osaka, Japan

**Keywords:** antibody-drug conjugate, circulating tumor DNA, clinical algorithm, metastasis-directed therapy, metastatic breast cancer, no evidence of disease, oligometastatic disease, tumor ecology

## Abstract

Metastatic breast cancer (MBC) has traditionally been managed as an incurable systemic disease in which the main goals are survival extension, symptom control, and preservation of quality of life. This principle remains appropriate for most patients. However, contemporary systemic therapies, high-resolution imaging, antibody-drug conjugates (ADCs), immune checkpoint inhibition, liquid biopsy, and metastasis-directed therapy (MDT) have made prolonged no evidence of disease (NED) clinically recognizable in selected patients. This Hypothesis and Theory article proposes NED-oriented oncology as a testable framework for carefully selected patients with MBC. The central premise is that complete response and NED should be separated conceptually: complete response is a radiological treatment-response category, whereas NED is a clinical state produced by the integration of systemic disease control, biological remodeling of the tumor immune microenvironment (TIME), and local eradication of all residual visible lesions. We propose a Clinical NED Score to structure candidate selection, a TIME-modifiability layer to estimate biological convertibility, and a five-step algorithm in which systemic therapy is used to generate deep response and immune-ecological remodeling before MDT is considered at maximal response. ADCs are discussed as potential tumor-ecosystem engineering tools because their effects may extend beyond direct cytotoxicity to bystander killing, immunogenic cell death, antigen release, and immune reconfiguration. This framework does not imply that MBC is broadly curable. Rather, it argues that durable NED is a clinically meaningful, prospectively testable state that should be distinguished from transient radiological response and evaluated using endpoints such as NED interval, polymetastatic progression-free survival, systemic therapy switch-free survival, ctDNA-negative duration, and quality-of-life preservation.

## 1 Introduction

Metastatic breast cancer (MBC) has historically been framed as a chronic but incurable systemic condition. This framing is clinically important because it prevents unrealistic promises, discourages excessive local intervention, and prioritizes survival, symptom control, and quality of life. At the same time, the meaning of metastatic disease is not fixed. Population-level analyses suggest that advances in screening and treatment have contributed substantially to reductions in breast cancer mortality, and international guidelines increasingly reflect a treatment landscape in which long-term control is possible for molecularly selected patients (1–3). The clinical challenge is therefore no longer limited to choosing the next systemic therapy; it also includes identifying whether a particular disease state can be converted into a durable state in which no active disease is detectable.

The therapeutic heterogeneity of MBC has expanded rapidly. Anti-HER2 therapy has transformed HER2-positive MBC from a historically aggressive phenotype into a subtype in which deep and durable responses are familiar in practice (4, 5). Trastuzumab deruxtecan has extended ADC efficacy into HER2-positive, HER2-low, and HER2-ultralow settings (5–7). Sacituzumab govitecan and datopotamab deruxtecan have broadened the relevance of ADCs across triple-negative and hormone receptor-positive/HER2-negative disease (8–10). Immune checkpoint inhibitors have improved outcomes in immunologically selected triple-negative disease (11, 12), whereas endocrine-based targeted therapy has produced prolonged disease control in hormone receptor-positive disease (13, 14). These advances create scenarios in which oligometastatic disease, oligoprogression, radiological complete response, and local consolidation are no longer rare anecdotes but recurring multidisciplinary questions.

This article proposes NED-oriented oncology as a hypothesis-generating framework for selected patients with MBC. The goal is not to claim that metastatic breast cancer is generally curable. The goal is to define when durable no evidence of disease (NED) is a rational treatment target, how candidate patients can be enriched, and how systemic therapy, tumor immune microenvironment (TIME) assessment, and metastasis-directed therapy (MDT) might be integrated into a testable clinical algorithm.

## 2 Why complete response and NED should be separated

**Figure 1:**
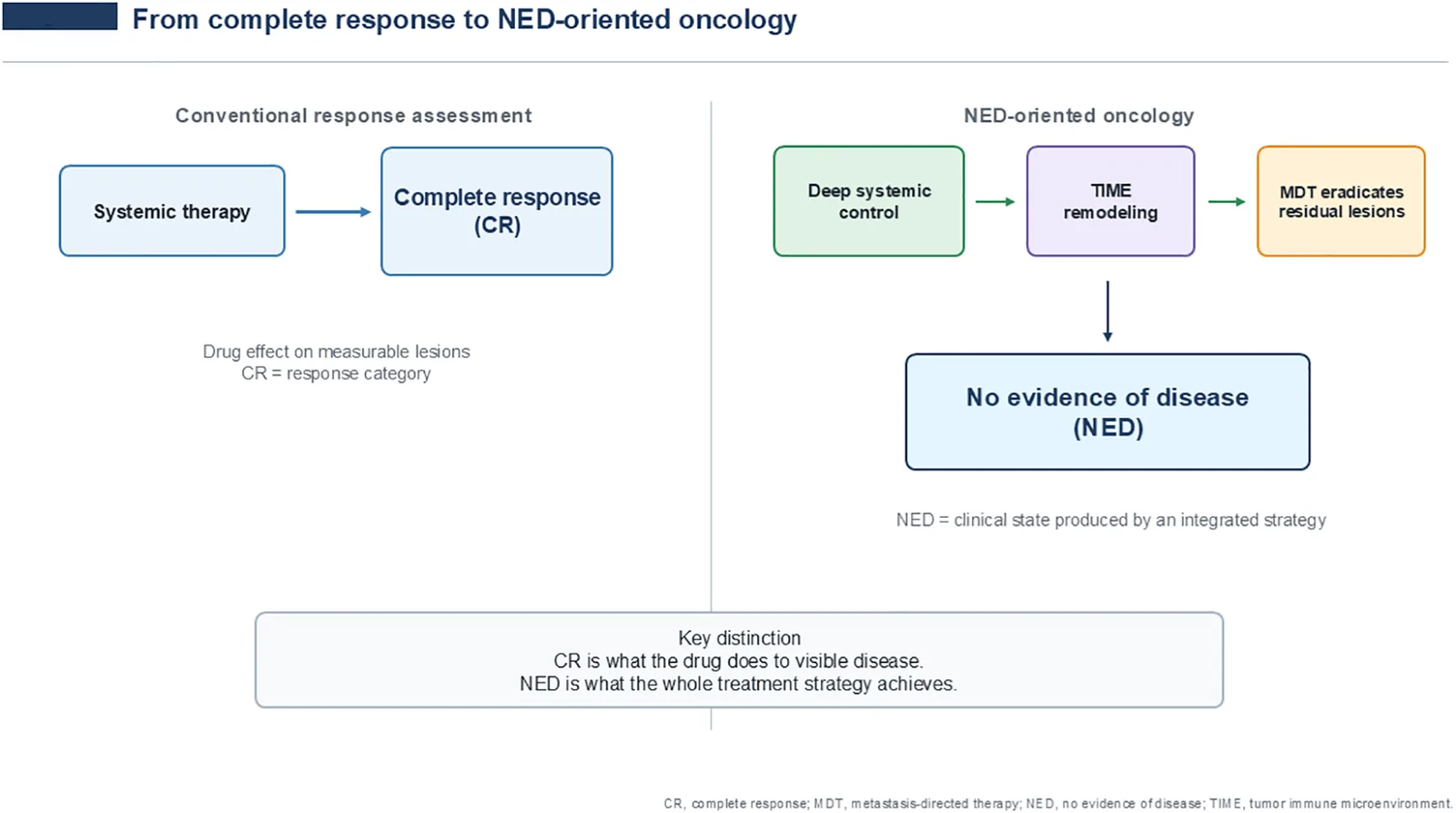
Conceptual distinction between complete response and NED. Complete response is a treatment-response category that describes disappearance of measurable disease after systemic therapy. NED is a clinically engineered state that can arise from systemic control, TIME remodeling, and eradication of residual visible lesions by MDT. The image frames the transition from response assessment to disease-state engineering.

Complete response (CR) and NED are often used interchangeably in clinical discussion, but they describe different concepts. CR is a response category, most commonly assessed by imaging criteria such as RECIST (15, 16). It describes disappearance of measurable target lesions after treatment. NED is a clinical state. It may be achieved through systemic therapy alone, systemic therapy followed by surgery, stereotactic body radiotherapy (SBRT), ablation, or combinations of local modalities. CR describes what a drug has done to visible disease; NED describes what the entire treatment strategy has achieved across the patient. This conceptual distinction is illustrated in **Figure 1**.

This distinction is not semantic. A patient may have a radiological CR but rapidly relapse through occult polymetastatic disease. Conversely, a patient may have an excellent partial response with one residual lesion that can be completely ablated, thereby reaching clinical NED. In long-term responders to first-line HER2-targeted therapy, patients who achieved NED had better progression-free and overall survival than those with persistent residual disease, although selection bias and favorable biology must be considered (17). Radiological complete response after first-line anti-HER2 therapy has also been associated with clinicopathological features that may identify candidates for future de-escalation or discontinuation studies (18). These observations support the concept that NED is clinically meaningful, but they do not establish NED as equivalent to cure.

We define NED, for the purpose of this framework, as a clinically engineered state in which no active disease is detectable by available clinical methods, all previously visible disease has either disappeared or has been locally eradicated, there is no evidence of uncontrolled polymetastatic dissemination, and the patient remains able to continue surveillance, maintenance therapy, or de-escalation strategies without unacceptable burden. This definition is intentionally conservative and should be communicated carefully to patients.

Operationally, NED should be confirmed using a protocol-defined assessment set, including contrast-enhanced CT and/or FDG-PET/CT when appropriate, site-specific MRI for brain or equivocal bone disease, tumor markers when clinically informative, and ctDNA when available. Equivocal lesions should prompt short-interval confirmation, additional imaging, or biopsy when feasible rather than immediate classification as durable NED; discordant radiological and molecular findings should be recorded separately in prospective studies. In prospective trials or registries, the assessment interval and the minimum duration of disease invisibility required to define durable NED should be specified a priori.

## 3 From subtype-based oncology to disease-state engineering

Breast cancer subtyping remains the entry point for therapeutic selection. Intrinsic molecular classifications and immunohistochemical surrogates based on estrogen receptor, progesterone receptor, HER2, and proliferation have shaped breast cancer oncology for more than two decades (19–21). However, receptor-based subtyping was designed primarily to classify disease and select drug classes. It was not designed to determine whether metastatic disease can be converted into durable NED.

The limitations of static subtype allocation are most apparent in modern MBC. Within the same subtype, some patients experience rapid polymetastatic progression whereas others achieve deep response, prolonged control, and locally consolidatable residual disease. Triple-negative breast cancer (TNBC) illustrates this problem. TNBC includes basal-like, immunomodulatory, mesenchymal, luminal androgen receptor, and other functional states rather than a single biological entity (22–25). Spatial and single-cell studies have further demonstrated that breast cancers are not uniform masses but structured ecosystems composed of tumor clones, immune cells, stromal barriers, vascular niches, and organ-specific metastatic contexts (26–29). These data suggest that receptor expression identifies therapeutic entry points, whereas NED potential depends on dynamic disease-state features.

NED-oriented oncology therefore asks a different question from conventional subtype-based treatment. Instead of asking only which systemic therapy matches a subtype, it asks whether the current disease state can be reshaped into one in which all visible lesions are eliminated and systemic control is maintained. This requires integrating four domains: clinical controllability, treatment sensitivity, local controllability, and biological modifiability.

Importantly, this framework should not be extrapolated uniformly across breast cancer subtypes. The clinical precedent for deep response and NED-like states is strongest in HER2-positive disease treated with effective anti-HER2 therapy; in HR-positive/HER2-negative disease, prolonged pharmacologic control and NED conversion should be distinguished carefully, particularly after endocrine resistance; and in TNBC, NED-oriented strategies require especially cautious selection based on response depth, disease kinetics, PD-L1/immune context, and early relapse risk.

## 4 Oligometastatic disease is necessary but not sufficient

The oligometastatic paradigm provides an essential foundation for NED-oriented oncology. Hellman and Weichselbaum proposed that some patients occupy an intermediate state between localized disease and widespread dissemination (30, 31). Subsequent ESTRO/EORTC and ASTRO/ESTRO efforts emphasized that oligometastatic disease should be defined not only by lesion number but also by disease history, imaging quality, treatment feasibility, and likelihood that all active sites can be controlled (32, 33). The SABR-COMET randomized phase II trial showed that stereotactic ablative radiotherapy added to standard palliative treatment improved survival in patients with controlled primary tumors and one to five metastases across multiple tumor types, although breast cancer-specific conclusions were limited by tumor heterogeneity and sample size (34, 35).

Breast cancer-specific consensus recommendations have reinforced this caution. The EORTC Imaging and Breast Cancer Groups highlighted the need for modern imaging, MDT feasibility, breast cancer subtype consideration, and prospective trial design in oligometastatic breast cancer (36). This is clinically important because lesion count alone can be misleading. A patient with several technically treatable lung or nodal lesions and slow disease kinetics may be more NED-eligible than a patient with two rapidly progressive liver lesions and rising tumor markers.

The CURB oligoprogression trial further demonstrated that SBRT improved progression-free survival in non-small-cell lung cancer but not in the breast cancer subgroup (37). This result should not be interpreted as evidence that MDT has no role in breast cancer. Rather, it suggests that oligoprogression in breast cancer contains biologically different states: isolated clonal escape during otherwise effective systemic therapy, sanctuary-site progression, mixed resistance, and early polymetastatic failure. MDT is rational when progression remains locally controllable while systemic control is preserved; it is not rational simply because the lesion count is small. Prospective breast cancer studies and institutional series continue to suggest that selected patients may have long disease-control intervals after local treatment, but selection remains the central issue (38, 39).

## 5 Proposed clinical NED score

To make NED-oriented oncology testable, candidate selection must be explicit. We propose the Clinical NED Score as a pragmatic, hypothesis-generating tool to structure multidisciplinary discussion. It is not a validated score and should not be used as a stand-alone decision instrument. Its purpose is to standardize the clinical question: is this patient currently in a disease state that could reasonably be converted to NED without sacrificing quality of life?

Accordingly, the Clinical NED Score should be interpreted as a consensus-generating framework for tumor-board discussion rather than as a validated treatment-selection tool. It is intended to make assumptions explicit, identify reasons for or against local consolidation, and define variables for prospective registries.

**Figure 2:**
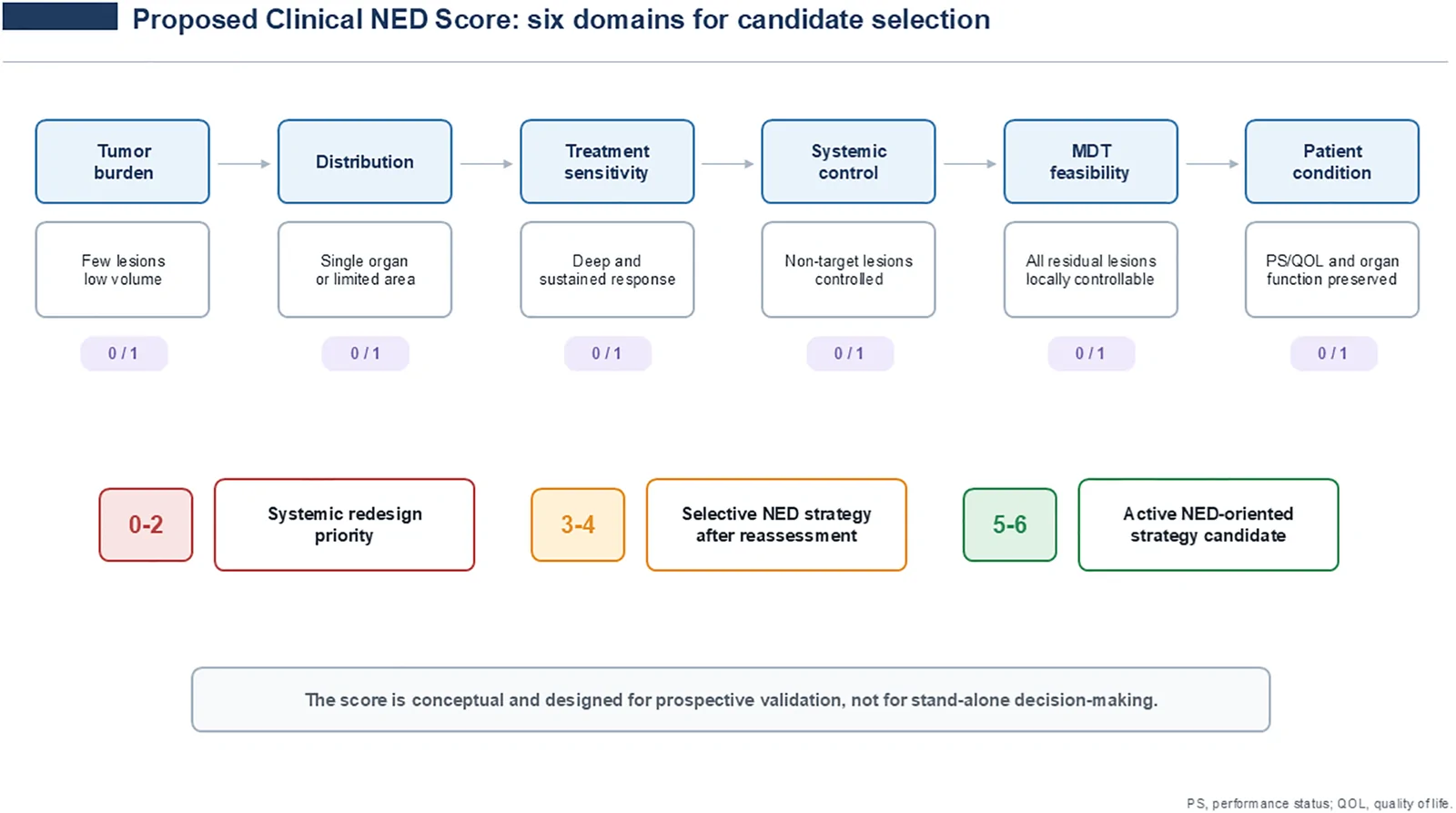
Proposed Clinical NED Score. Six clinical domains are used to identify patients who may be candidates for a NED-oriented strategy: tumor burden, anatomical distribution, treatment sensitivity, systemic control, MDT feasibility, and patient condition. The score is conceptual and requires prospective validation.

The score assigns one point for each of six favorable domains: low tumor burden, limited anatomical distribution, deep or sustained systemic treatment sensitivity, preserved systemic control of non-target lesions, feasibility of complete local control by surgery, SBRT, ablation, or other MDT, and adequate patient condition including performance status, organ function, patient preference, and quality of life. A score of 5-6 suggests that an active NED-oriented strategy may be discussed; 3-4 suggests selective consideration after additional systemic treatment or reassessment; 0-2 suggests that systemic redesign and disease control should be prioritized. The proposed Clinical NED Score and its six domains are summarized in **Figure 2** and **Table 1**.

**Table 1:** Proposed clinical NED score for selected patients with metastatic breast cancer.

Domain	Favorable feature	Unfavorable feature	Score
Tumor burden	Few visible lesions with low total disease volume and no rapidly enlarging bulky lesion	Multiple bulky lesions, rapidly increasing tumor burden, or disease volume incompatible with complete local consolidation	0/1
Distribution	Single organ, limited anatomical region, or anatomically consolidatable sites	Diffuse multi-organ, peritoneal, leptomeningeal, miliary pulmonary, or otherwise non-consolidatable pattern	0/1
Treatment sensitivity	Early, deep, and sustained response; tumor marker or ctDNA decline when available	Mixed response, shallow response, new lesions, rising markers, persistent ctDNA, or resistant disease kinetics	0/1
Systemic control	Non-target lesions controlled and current systemic therapy can continue	Systemic treatment failure or inability to maintain therapy	0/1
MDT feasibility	All residual lesions safely controllable by surgery, SBRT, ablation, or combined MDT after tumor-board review	Residual disease not technically or safely controllable	0/1
Patient condition	Preserved PS, QOL, organ function, and patient preference compatible with a NED-oriented strategy	Poor PS, organ dysfunction, or QOL burden outweighing benefit	0/1

Equal weighting is used only for simplicity at the hypothesis-generating stage. Future validation should determine whether weights should differ by subtype, line of therapy, endocrine resistance, prior ADC exposure, organ site, performance status, and patient preference.

The score is intentionally dynamic. A patient may initially have high-volume disease and a low score but later become a candidate after systemic therapy reduces disease to a few residual sites. Conversely, an initially oligometastatic patient may lose eligibility if mixed response, rapid new-lesion emergence, persistent ctDNA, or declining organ function develops. The score should be repeated at diagnosis of metastatic disease, early response assessment, maximal response, oligoprogression, and post-MDT surveillance.

For example, a patient with HER2-positive MBC who achieves deep systemic response with one safely ablatable residual liver lesion, controlled non-target disease, declining markers/ctDNA, and preserved QOL may score 5-6 and be discussed for MDT at maximal response. In contrast, a patient with endocrine-resistant HR-positive/HER2-negative disease, mixed hepatic response, new bone lesions, rising ctDNA, and declining organ function should be managed as systemic treatment failure even if only a few lesions are measurable.

## 6 TIME as the biological layer of NED potential

Clinical controllability and biological controllability are not identical. Patients with similar tumor burden and lesion distribution may have different outcomes because the TIME determines whether residual disease is immunologically suppressed, excluded, or primed for elimination. Tumor-infiltrating lymphocytes (TILs) remain the most accessible clinical window into TIME. International recommendations have standardized TIL assessment, and higher TILs have prognostic and predictive relevance particularly in TNBC and HER2-positive disease (40–44). However, TILs are not static. Immune architecture varies between primary tumors and metastases, across organ sites, and after therapy exposure.

Single-cell, spatial transcriptomic, and high-dimensional imaging studies have shifted TIME interpretation from cell abundance to spatial organization and functional state (26–29, 45, 46). A tumor may be immune-inflamed, immune-excluded, immunosuppressed, or immune-desert; each state has different implications for ICI, chemotherapy, ADCs, and MDT. General cancer immunology frameworks such as the cancer-immunity cycle and immune contexture also support the idea that immune control is spatial, temporal, and treatment-modifiable rather than a fixed binary status (47–50).

**Figure 3:**
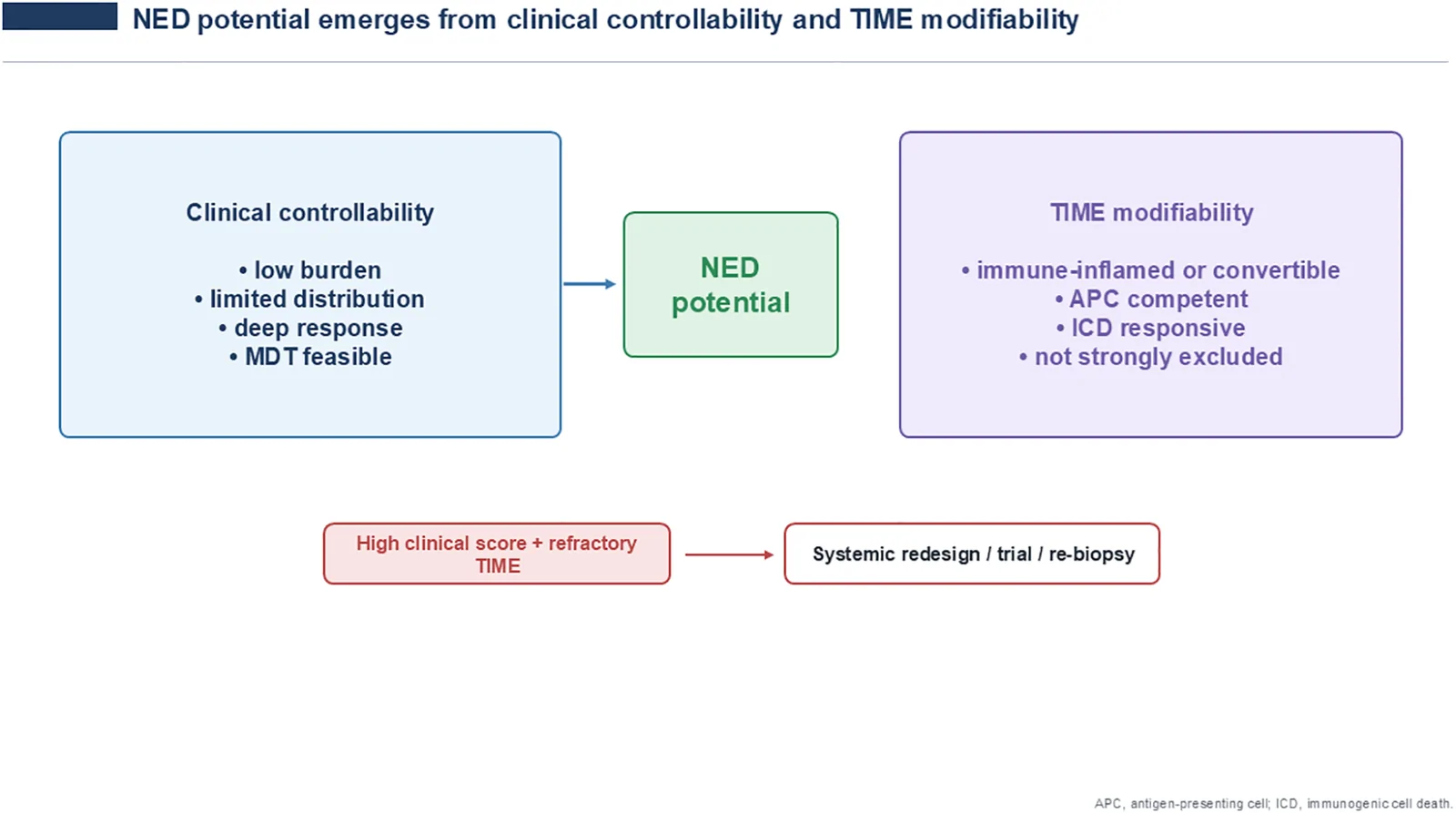
Integration of clinical controllability and TIME modifiability. NED potential emerges when clinically controllable disease overlaps with a favorable or modifiable TIME. High clinical controllability with refractory TIME should prompt systemic redesign, clinical trial consideration, repeat biopsy, or closer surveillance before MDT.

For clinical translation, we propose three broad TIME categories. A favorable TIME shows immune-inflamed architecture, cytotoxic T-cell infiltration, antigen-presentation competence, and absence of dominant exclusion barriers. A modifiable TIME is initially suboptimal but shows features suggesting that therapy could induce immunogenic cell death, antigen release, myeloid repolarization, vascular normalization, or stromal remodeling. A refractory TIME is characterized by immune desert or exclusion, poor antigen presentation, suppressive myeloid niches, dense stroma, hypoxia, or persistent molecular disease despite radiological response. The overlap between clinical controllability and TIME modifiability is the biological core of NED potential. The interaction between clinical controllability and TIME modifiability is summarized in **Figure 3**.

## 7 ctDNA and molecular invisibility

NED is currently a clinical and radiological state, but molecular residual disease may refine its meaning. Circulating tumor DNA (ctDNA) can detect molecular relapse in early breast cancer and can monitor tumor burden, resistance evolution, and treatment response in advanced disease (50–53). In a NED-oriented framework, ctDNA clearance may provide a molecular correlate of disease invisibility, whereas persistent ctDNA after apparent radiological control may identify biologically fragile NED.

However, ctDNA interpretation must be cautious. Shedding varies by tumor biology, metastatic site, volume, assay design, and treatment context. Low-volume or indolent disease may be ctDNA-negative even when viable disease persists. Conversely, transient ctDNA positivity during treatment-induced tumor cell death may not always indicate progression. Therefore, ctDNA should be integrated with imaging, tumor markers, biopsy when feasible, and clinical kinetics rather than treated as a stand-alone gatekeeper. Future NED-oriented trials should prospectively evaluate ctDNA-negative duration, molecular relapse patterns, and whether ctDNA-guided MDT or systemic redesign improves outcomes.

## 8 ADCs as tumor-ecosystem engineering tools

ADCs are often described as targeted chemotherapy, but this description underestimates their biological complexity. Modern ADCs integrate antigen binding, antibody internalization, linker stability, payload release, membrane permeability, bystander killing, Fc-mediated immune interactions, and payload-dependent cell death programs (54–59). In breast cancer, ADCs have already transformed HER2-positive, HER2-low, HER2-ultralow, TNBC, and hormone receptor-positive/HER2-negative therapeutic landscapes (5–10).

**Figure 4:**
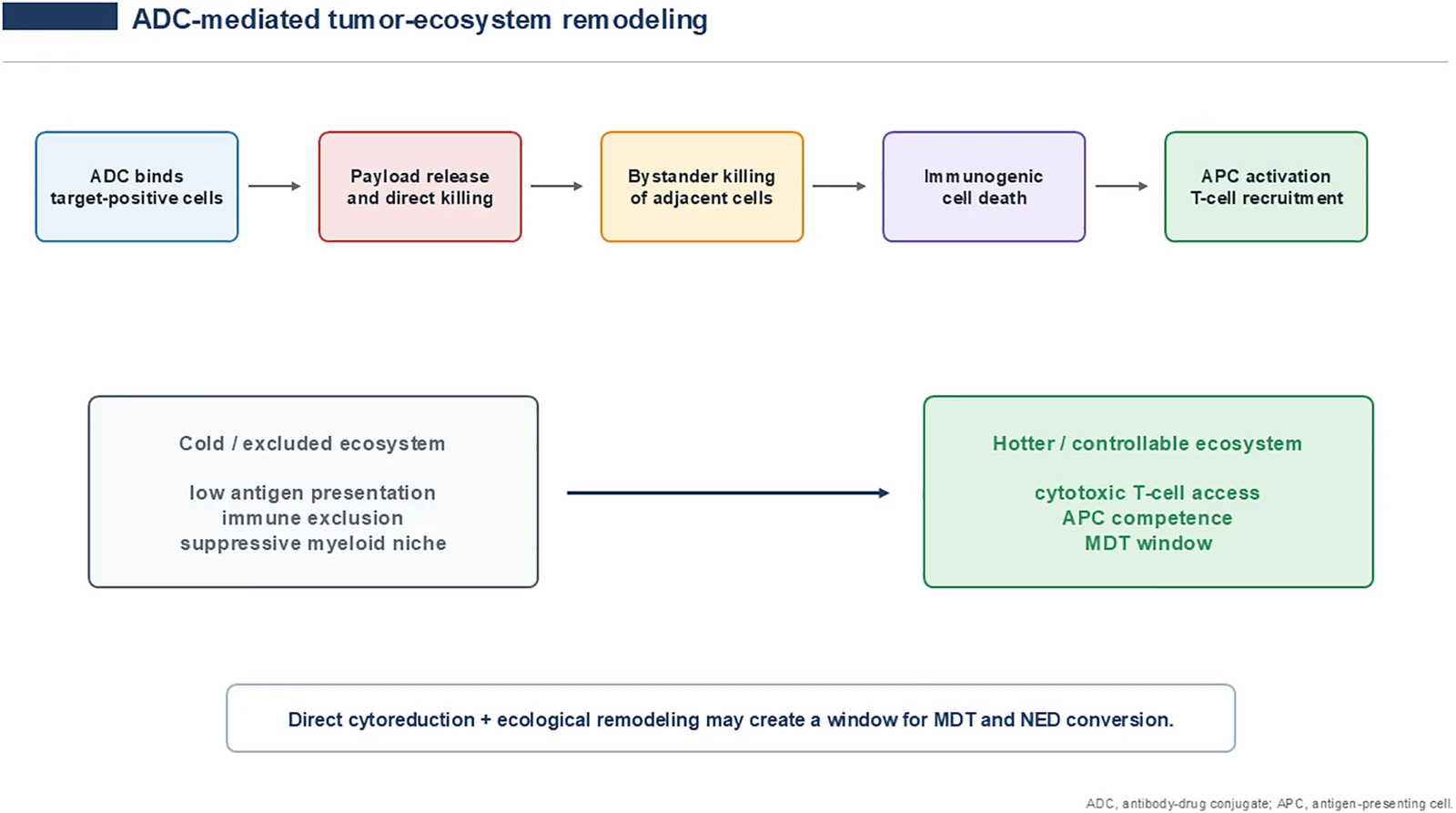
ADC-mediated tumor-ecosystem remodeling. Modern ADCs may contribute to NED conversion through direct tumor cell killing, bystander killing, immunogenic cell death, antigen release, antigen-presenting-cell activation, and immune reconfiguration. These effects depend on target distribution, linker-payload properties, drug penetration, and pre-existing TIME.

The relevance of ADCs to NED-oriented oncology is twofold. First, ADCs can produce deep cytoreduction, converting disseminated disease into limited residual disease. Second, selected ADCs may remodel TIME. Preclinical work with trastuzumab deruxtecan and related platforms shows that linker-payload properties, extracellular payload release, and bystander killing can shape therapeutic effects in heterogeneous tumors (60–63). Recent experimental data suggest that trastuzumab deruxtecan may also induce immunomodulatory interactions involving antigen release, myeloid antigen-presenting cells, and T-cell responses (62). Sacituzumab govitecan has demonstrated bystander killing in heterogeneous Trop-2-expressing models and Fc-associated immune effects in preclinical systems (63). These mechanisms connect ADC therapy to ecological remodeling rather than direct killing alone. These proposed mechanisms of ADC-mediated tumor-ecosystem remodeling are summarized in **Figure 4**.

These statements should be read as a biological rationale for prospective testing rather than as evidence that ADCs reliably induce clinically meaningful immune memory or durable NED in current practice.

This does not mean that all ADCs are equivalent TIME-remodeling agents. Immune consequences likely depend on payload class, linker stability, membrane permeability, antigen density, spatial antigen distribution, drug penetration, Fc function, extracellular protease activity, organ microenvironment, and prior therapy. Immunogenic cell death also depends on context: anthracyclines, oxaliplatin, radiotherapy, and some targeted agents can induce danger signals such as calreticulin exposure, ATP release, and HMGB1 release, but whether a specific ADC produces clinically meaningful immune memory must be tested rather than assumed (64–66). The NED-oriented question is therefore not simply whether the target is present; it is whether the ADC can generate deep response and ecological remodeling sufficient to create a window for MDT.

## 9 MDT as the finishing step, not the starting point

In NED-oriented oncology, MDT should usually be regarded as a finishing step after systemic therapy has demonstrated disease controllability. This differs from applying local therapy reflexively whenever a small number of lesions are visible. The therapeutic logic is sequential: systemic therapy tests metastatic biology, reduces disease burden, and may remodel TIME; maximal response reveals whether residual disease is locally controllable; MDT eradicates residual visible lesions; and surveillance or maintenance therapy preserves NED.

Before MDT is performed, four questions should be answered in a multidisciplinary tumor board. First, are all active lesions known and technically controllable? Second, is systemic therapy controlling non-target disease? Third, does biology suggest local escape or residual limited disease rather than generalized systemic failure? Fourth, does the expected benefit justify procedural risk and patient burden? When these conditions are met, surgery, SBRT, ablation, or combined local modalities may transform deep response into NED. When these conditions are not met, MDT may delay necessary systemic redesign and expose the patient to futile morbidity.

This framework is also relevant to oligoprogression. A progression event should be categorized as locally controllable progression, sanctuary-site progression, mixed resistance, or polymetastatic failure. Only the first two categories are likely to benefit from MDT while continuing an otherwise effective systemic regimen. This distinction is central to trial design because conventional PFS treats all progression events similarly, whereas NED-oriented oncology depends on the clinical meaning of the progression event.

The evidence base for MDT should therefore be interpreted cautiously. Apparent benefit may reflect patient selection, favorable disease biology, immortal-time bias among patients who survive long enough to receive MDT, and selection of lesions that are technically easy to eradicate; toxicity, cost, and QOL trade-offs should be prospectively captured.

## 10 Five-step algorithm

**Figure 5:**
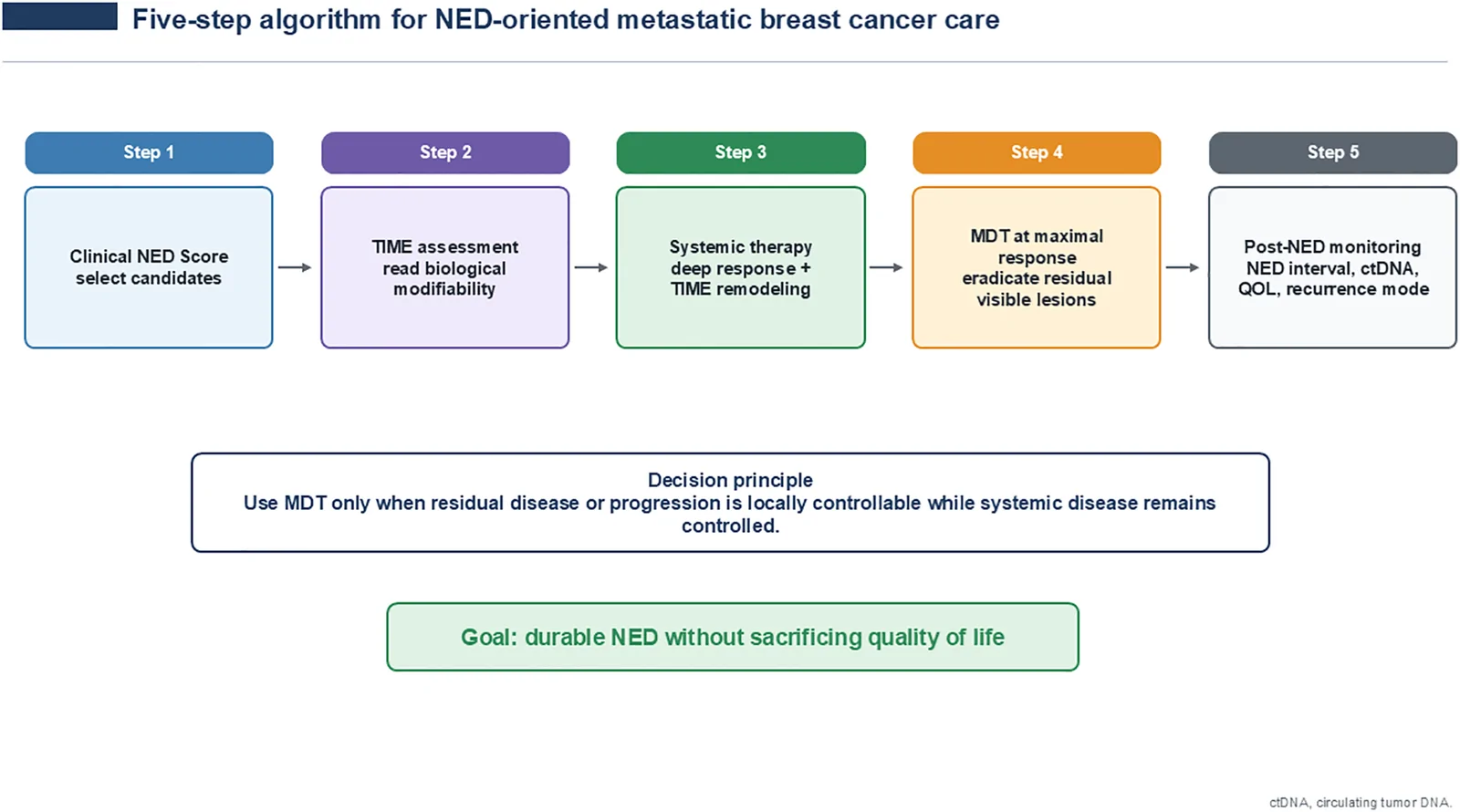
Five-step algorithm for NED-oriented metastatic breast cancer care. The proposed algorithm begins with candidate selection, adds biological assessment of TIME, uses systemic therapy capable of deep response and TIME remodeling, applies MDT at maximal response when all residual disease is locally controllable, and follows patients using NED-specific endpoints.

We propose a five-step algorithm for selected patients with MBC. Step 1 is candidate selection using the Clinical NED Score. Step 2 is biological assessment of TIME modifiability using the most accessible tools available in each setting: subtype, TILs, metastatic biopsy when feasible, tumor markers, ctDNA, response kinetics, and, in research settings, multiplex immunohistochemistry, spatial transcriptomics, single-cell profiling, and immune repertoire analysis. Step 3 is systemic therapy aimed at deep response and TIME remodeling. Depending on subtype and treatment line, this may involve anti-HER2 therapy, endocrine therapy plus targeted agents, ICI-based regimens, ADCs, chemotherapy, PARP inhibitors, AKT/PI3K pathway inhibitors, or clinical trials. Step 4 is MDT at maximal response only when all residual disease is locally controllable and systemic control remains preserved. Step 5 is post-NED monitoring using imaging, clinical assessment, tumor markers, ctDNA where available, toxicity assessment, and patient-reported quality of life. The overall five-step workflow is summarized in **Figure 5**.

The algorithm is not intended to replace guidelines. It is a decision architecture for tumor boards and clinical trials. Its value lies in preventing two errors: undertreating patients who may be convertible to durable NED, and overtreating patients whose apparent oligometastatic status masks systemic failure. The algorithm also provides a structure for prospective registries in which patients can be scored, profiled, treated, locally consolidated, and followed using NED-specific endpoints.

## 11 Endpoints for NED-oriented trials

PFS and OS remain essential endpoints, but PFS alone is insufficient to evaluate NED-oriented strategies. In conventional PFS analysis, any progression event is counted similarly. In NED-oriented oncology, a new solitary locally treatable lesion under preserved systemic control differs fundamentally from diffuse polymetastatic spread requiring systemic therapy change.

**Figure 6:**
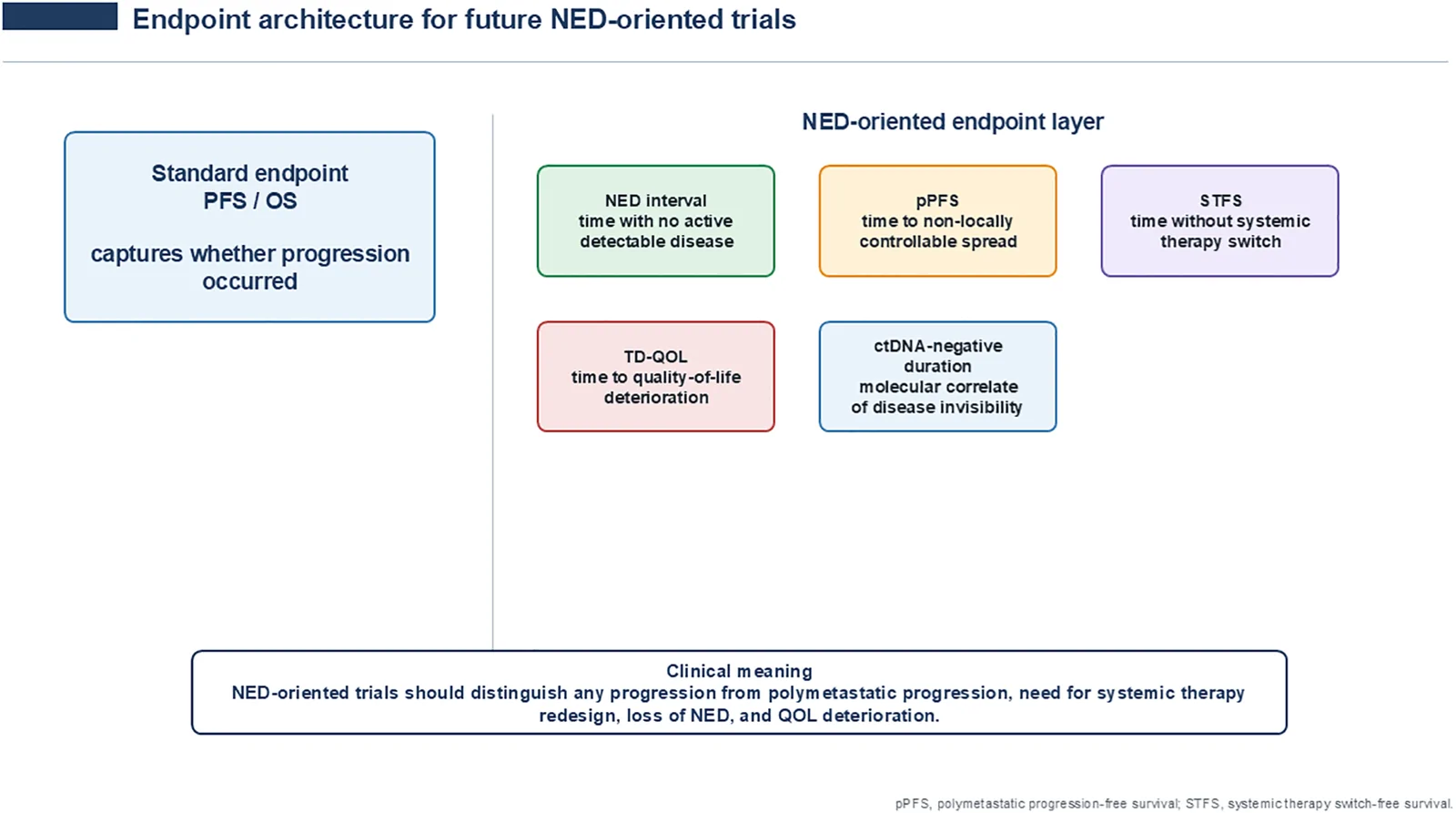
Endpoint architecture for future NED-oriented trials. PFS alone does not fully capture the value of NED-oriented strategies. Complementary endpoints include NED interval, polymetastatic progression-free survival, systemic therapy switch-free survival, time to quality-of-life deterioration, and ctDNA-negative duration.

Complementary endpoints should therefore be incorporated into future trials. NED interval measures the time during which no active disease is detectable. Polymetastatic progression-free survival measures time to non-locally controllable spread. Systemic therapy switch-free survival measures whether MDT can defer systemic therapy redesign. Time to deterioration of quality of life measures whether systemic-local intensification preserves the patient’s lived experience. ctDNA-negative duration may provide a molecular correlate of NED, although it requires assay-specific validation. Patient-reported outcomes are particularly important because a NED-oriented strategy that worsens quality of life without extending meaningful control would not be justified (67, 68). The proposed endpoint architecture is illustrated in **Figure 6**, and the definitions and clinical rationale for these endpoints are summarized in **Table 2**

**Table 2:** Candidate endpoints for future NED-oriented trials.

Endpoint	Definition in NED-oriented trials	Why it matters
NED interval	Time with no clinically detectable active disease	Captures duration of the intended clinical state
Polymetastatic PFS	Time to non-locally controllable metastatic spread	Distinguishes treatable oligoprogression from systemic failure
Systemic therapy switch-free survival	Time without need to start or change systemic therapy	Measures whether MDT preserves an effective regimen
Time to QOL deterioration	Time to clinically meaningful decline in patient-reported QOL	Prevents overtreatment that harms lived experience
ctDNA-negative duration	Duration of molecular non-detectability using a defined assay	Provides a molecular correlate of disease invisibility

For clinical-trial translation, each endpoint should include prespecified estimand language. Repeat MDT for isolated oligoprogression, systemic therapy change for toxicity rather than progression, ctDNA positivity without radiological disease, treatment discontinuation after NED, and death without prior progression should be counted, censored, or reported as separate intercurrent events according to the trial objective.

Adaptive trial designs may be most suitable. Patients with high Clinical NED Scores and favorable or modifiable TIME could receive optimized systemic therapy followed by protocol-defined MDT at maximal response. Randomization could occur before MDT, after systemic response, or after NED achievement depending on the hypothesis. Translational endpoints should evaluate whether durable NED is mediated by immune activation, clonal extinction, stromal remodeling, continued pharmacologic suppression, or a combination of these processes.

## 12 Testable hypotheses

The framework yields several testable hypotheses. First, the Clinical NED Score will enrich for patients who achieve NED after systemic therapy with or without MDT. Second, adding TIME features will improve prediction beyond clinical features alone. Third, ADC-induced conversion to limited residual disease will be associated with immune activation, antigen-presentation enhancement, spatial immune reorganization, or ctDNA clearance in patients who achieve durable NED. Fourth, MDT performed after deep systemic response will prolong NED interval and systemic therapy switch-free survival in selected patients compared with continued systemic therapy alone. Fifth, patients who relapse after NED will show distinct failure modes: isolated local relapse amenable to repeat MDT, oligoprogression under preserved systemic control, sanctuary-site escape, or polymetastatic progression requiring systemic redesign.

These hypotheses can be tested initially through prospective registries. Such registries should capture baseline disease distribution, subtype, prior therapy, systemic treatment response, tumor markers, ctDNA, metastatic biopsy features, TILs or multiplex immune profiling, MDT details, adverse events, quality of life, NED interval, systemic therapy changes, and mode of progression. Registry data can refine the Clinical NED Score, identify which biological layers add predictive value, and support randomized trials.

## 13 Limitations and safeguards

The major risk of NED-oriented oncology is overstatement. Durable NED is not synonymous with cure, and the framework should not be presented to patients as a promise of curability. The term NED-oriented should mean a disciplined strategy to identify exceptional disease-control states and test whether they can be reproduced, not a claim that metastatic disease can be eradicated in most patients.

A second limitation is selection bias. Patients who reach NED often have favorable biology, low disease burden, better performance status, access to advanced imaging, and more opportunities for multidisciplinary care. Retrospective comparisons between NED and residual disease may therefore overestimate the causal effect of NED itself. Prospective studies must determine whether NED is a mediator of improved outcome, a marker of favorable biology, or both.

This concern is especially relevant when comparing patients who achieved NED with those who did not, because NED may be both a mediator of outcome and a marker of favorable biology.

A third limitation is feasibility. TIME profiling, spatial transcriptomics, serial biopsies, and ctDNA monitoring are not universally available. For this reason, the framework uses a layered strategy: clinical scoring for broad use, TILs and ctDNA where available, and advanced spatial-immune profiling in translational trials. Finally, MDT has procedural risks. Surgery, SBRT, and ablation should be offered only when morbidity is justified by realistic disease-control goals and patient preference.

## 14 Discussion and conclusion

The management of breast cancer has moved from histology to receptor status, from receptor status to molecular subtyping, and now toward dynamic tumor-ecosystem interpretation. MBC care should evolve accordingly. The central hypothesis of this article is that, in selected patients, the clinically meaningful question is not only which systemic therapy should be used next, but whether the disease state can be engineered toward NED.

NED-oriented oncology is not a replacement for subtype-based therapy. Subtype determines the therapeutic entrance. NED orientation determines whether and when to pursue conversion, consolidation, and durable disease invisibility. In this framework, ADCs are not merely late-line cytotoxic drugs; they may become agents of ecological remodeling. ICI is not merely a drug class; it is a therapy dependent on immune context. MDT is not merely local control; it is the final step in a systemic-local sequence.

The next challenge is validation. The Clinical NED Score should be prospectively tested, TIME layers should be standardized, and NED-specific endpoints should be embedded into trials of ADCs, ICI combinations, targeted therapy, and MDT. If validated, this framework could help clinicians identify patients for whom aggressive integration of systemic and local therapy is rational while protecting others from futile intervention. The purpose is not to replace caution with optimism, but to convert exceptional outcomes into reproducible clinical science.

## Data Availability

The original theoretical contributions presented in the article are included in the article. Further inquiries can be directed to the corresponding author.
